# Poor sleep quality in migraine and probable migraine: a population study

**DOI:** 10.1186/s10194-018-0887-6

**Published:** 2018-07-25

**Authors:** Tae-Jin Song, Soo-Jin Cho, Won-Joo Kim, Kwang Ik Yang, Chang-Ho Yun, Min Kyung Chu

**Affiliations:** 1 Department of Neurology, College of Medicine, Ewha Womans University, Seoul, South Korea; 2 Department of Neurology, Dongtan Sacred Heart Hospital, Hallym University College of Medicine, Hwaseong, South Korea; 3 Department of Neurology, Gangnam Severance Hospital, Yonsei University, College of Medicine, Seoul, South Korea; 4 Department of Neurology, Soonchunhyang University College of Medicine, Cheonan Hospital, Cheonan, South Korea; 5 Department of Neurology, Clinical Neuroscience Center, Seoul National University Bundang Hospital, Seongnam, South Korea; 6 Department of Neurology, Yonsei University College of Medicine, 50-1 Yonsei-ro, Seodaemoon-gu, Seoul, 03722 South Korea

**Keywords:** Headache, Migraine, Pittsburgh sleep quality index, Sleep, Sleep quality

## Abstract

**Background:**

Probable migraine (PM) is a subtype of migraine that is prevalent in the general population. Previous studies have shown that poor sleep quality is common among migraineurs and is associated with an exacerbation of migraine symptoms. However, information on the prevalence and clinical implication of poor sleep quality among individuals with PM is scarce. Thus, the aim of this study was to assess the prevalence and clinical impact of poor sleep quality in individuals with PM in comparison with those with migraine.

**Methods:**

Two-stage cluster random sampling was used to perform the survey for sleep and headache in Korean general population. Participants with Pittsburgh Sleep Quality Index > 5 were considered as having poor sleep quality.

**Results:**

Of 2695 participants, 379 (14.1%) had PM and 715 (26.5%) had poor sleep quality. Prevalence of poor sleep quality was 35.4% in the PM group, which was lower than that in the migraine group (47.6%, *p* = 0.011), but higher than that in the non-headache group (21.4%, *p* < 0.001). The PM participants with poor sleep quality showed increased headache frequency (median [interquartile range]: 2.0 [0.3–4.0] vs. 1.0 [0.2–2.0]; *p* = 0.001) and headache intensity (visual analogue scale, 6.0 [4.0–7.0] vs. 5.0 [3.5–6.0]; *p* = 0.003) compared to PM participants who had no poor sleep quality.

**Conclusions:**

Poor sleep quality was prevalent among participants with PM. It was associated with an exacerbation of PM symptoms. Our findings suggest that proper evaluation and treatment for poor sleep quality are needed in the management of PM.

**Electronic supplementary material:**

The online version of this article (10.1186/s10194-018-0887-6) contains supplementary material, which is available to authorized users.

## Background

Probable migraine (PM) is classified when one of diagnostic criteria of migraine in the third edition of the international classification of headache disorders (ICHD-3) is not applicable [1]. Probable migraine affects approximately 5–10% of the general population. It causes significant amount of disability owing to its symptoms such as migraine [2–4]. Although previous studies have demonstrated that patients with PM have relatively milder headache symptoms compared to individuals with migraine [3, 5], many patients with PM experience poor quality of life related to health with considerable disability [4].

Previous researches have revealed that sleep disturbances are frequent among migraineurs. Accompanying insomnia is more frequently noted in migraineurs than that in non-migraineurs [6]. Additionally, excessive daytime sleepiness is more prevalent among migraineurs. It is associated with worsening migraine symptoms [7]. Habitual snoring and bruxism are known to be risk factors of chronic migraine, a chronic form of migraine showing more severe symptoms and more frequent comorbidities compared to episodic migraine [8, 9]. Restless legs syndrome is also significantly associated with migraine [10].

Both sleep quantity and quality are important not only for health, but also for well-being [11]. Sleep studies have demonstrated that duration of sleep does not differ between non-migraineurs and migraineurs [12, 13]. Therefore, difference in sleep quality may explain the higher sleep disturbance in migraineurs. Several studies have shown that poor sleep quality in migraineurs is more frequently noted compared to that in non-migraine individuals with headache [14, 15]. Moreover, migraineurs who had poor sleep quality showed more frequent headache as well as symptoms of depression and anxiety [16].

Although PM is a common headache disorder, information about prevalence and clinical impact of poor sleep quality in individuals with PM is limited. We hypothesized that poor sleep quality would be prevalent in participants with PM and that it would be associated with an aggravation of clinical presentation of PM, as in the case with migraine. The Korean Headache-Sleep Study (KHSS) is a nationwide, general population-based survey about headache and sleep. The KHSS may give us a chance to investigate the relationship of PM with poor sleep quality. Thus, the aim of this study was to evaluate the prevalence and impact of poor sleep quality among participants with PM and those with migraine using data of the KHSS. Factors associated with poor sleep quality in participants with PM were also assessed.

## Methods

### Survey

KHSS is a nation-wide and cross-sectional study regarding headache and sleep characteristics among adult (19–69 years old) across Korean general population. Detailed protocol and methods of KHSS were described elsewhere [17]. Briefly, KHSS used two-stage random sampling methods. The study population of KHSS was based on the distribution of Korean population except Jeju-island [17]. To prevent interest bias, we informed all participants that our survey theme was a social health issue than neurological disorders such as headache and sleep problems. The survey was conducted by door-to-door visits and face-to-face interviews, using a structured questionnaire. The questionnaire covered information regarding characteristics of headache, sleep, anxiety, and depression. All interviewers were not medical related workers. They were members of Gallup Korea. The KHSS was approved by the Institutional Review Board of Hallym University Sacred Heart Hospital (approval No. 2011-I077). We received written consent from all participants before the survey interview. The survey was performed from November 2011 to January 2012.

### Diagnosis of migraine and PM

Migraine and PM were diagnosed based on ICHD-2 diagnostic criteria which was valid at that time [18]. Migraine was defined based on code 1.1 migraine without aura of ICHD-2 [18]. If a participant satisfied A, B, C, and D criteria of 1.1 migraine without aura, she/he was classified as having migraine. If one of criteria for migraine was not satisfied, the participant was classified as having PM. It is difficult to define code 1.2.1 migraine with aura or code 1.6.2 PM with aura in epidemiological study using questionnaire survey methods [19]. Therefore, our study did not estimate the presence of aura. Our study’s migraine included both code 1.1 migraine without aura and code 1.2 migraine with aura [18] while PM included both code 1.6.1 PM without aura and code 1.6.2 PM with aura [18]. Our study questionnaire exhibited a sensitivity of 75.0% and a specificity of 88.2% in migraine diagnosis compared to doctor’s diagnosis via telephone interview and result in the survey [20]. Frequency of headache per month, visual analogue scale (VAS) for headache intensity, and Headache Impact Test-6 (HIT-6) for impact of headache was investigated. Non-headache participants were defined as those who reported no headache during the previous year.

### Poor sleep quality, anxiety, and depression

Pittsburgh Sleep Quality Index (PSQI) was applied to assess sleep quality. Participants with total PSQI score > 5 were defined as having poor sleep quality [21]. We also investigated each component score of PSQI such as subjective quality of sleep, latency of sleep, duration of sleep, habitual sleep insufficiency, disturbance of sleep, use of hypnotics, and dysfunction at daytime [21]. Goldberg Anxiety Scale was used for the diagnosis of anxiety. The Goldberg Anxiety Scale includes four screening items and five supplementary items [22, 23]. Participants who presented positive answers with more than two of screening items or with more than five of all scale items were classified as persons with anxiety. The Korean version of Goldberg Anxiety Scale was validated in previous studies, with a sensitivity of 82.0% and a specificity of 94.4% [23, 24]. Patient Health Questionnaire-9 (PHQ-9) was applied to investigate the presence of depression [25]. In this scoring system, presence of depression was defined as 10 points or more. The Korean version of PHQ-9 was also validated with a sensitivity of 81.1% and a specificity of 89.9% [26].

### Statistical analyses

All statistical analyses were performed using SPSS 22.0 (IBM, Armonk, NY, USA). Kolmogorov-Smirnov test was used to check normal distribution for continuous variables. If variables showed normal distribution, independent t-test or one-way analysis of variance was used. If variables did not show normal distribution, Mann-Whitney U-test or Kruskal-Wallis test was performed. Categorical variables were analysed using Chi-square test or Fisher’s exact test. We used Mann−Whitney *U*-test to compare headache frequency per month, VAS score for headache intensity, and HIT-6 score between participants had PM with and without poor sleep quality.

For assessing factors contributing to poor sleep quality among participants with PM, we performed multivariable linear regression analyses after adjusting sociodemographic variables (age, sex, residential area size, and level of education), GAS score for anxiety, PHQ-9 score for depression, frequency of headache (per month), and intensity of headache (VAS score). Statistical significance was considered when *p* value (two-tailed) was less than 0.05.

## Results

### Survey

**Fig. 1:**
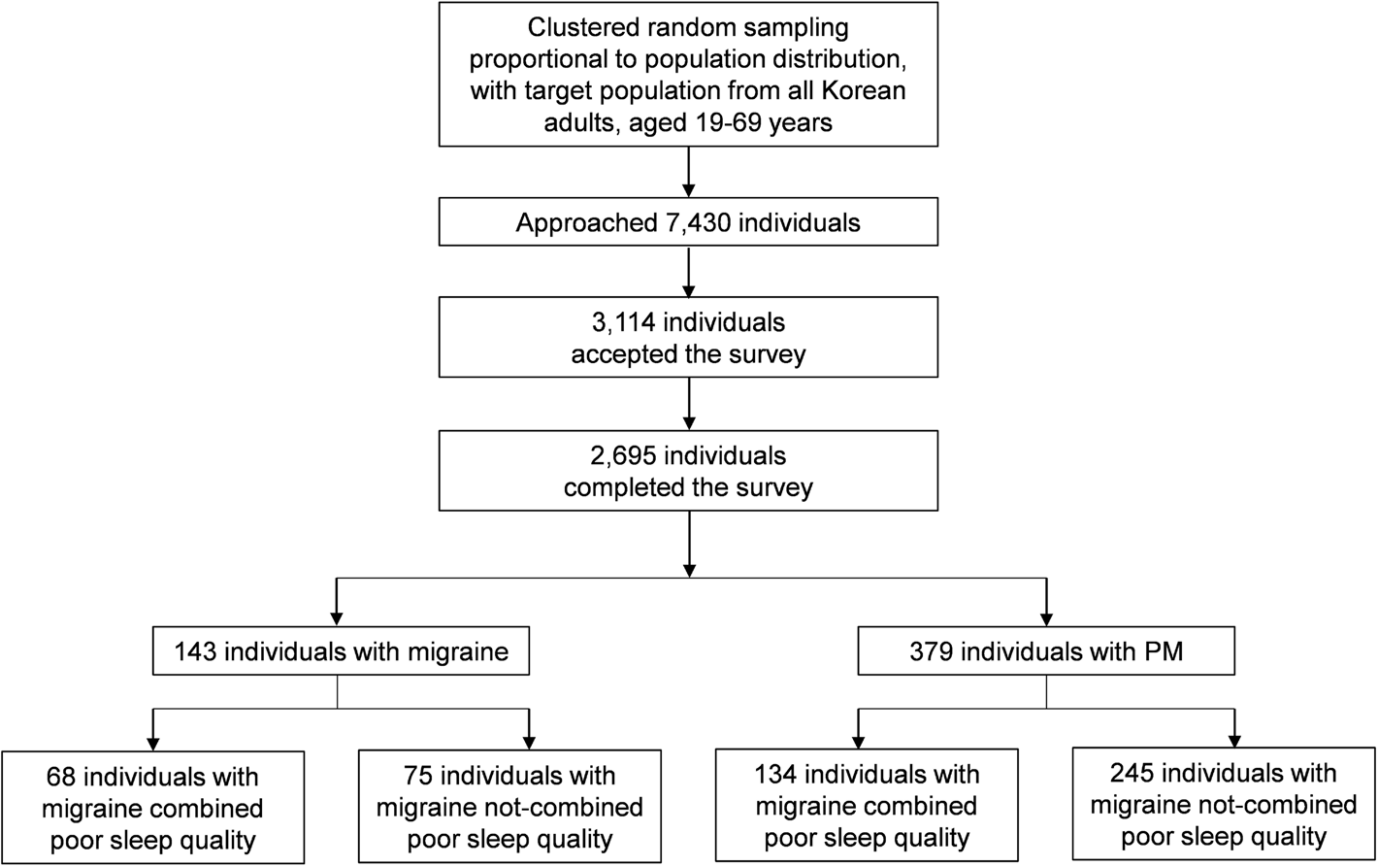
Flowchart depicting enrolment and participation in the Korean Headache-Sleep Study

We interviewed 7430 people and 3114 people who agreed to participate in our study (58.1% of rejection rate). Of these, 419 people withdraw participation during the interview. Finally, 2695 people completed our survey (36.3% of cooperation rate, Fig. 1). Distribution of sex, age, residence size, or education level of our sample was not significantly different from that in the general population in Korea (Table 1).

**Table 1:** Sociodemographic characteristics of survey participants, the total Korean population, and cases identified as having migraine, probable migraine, and poor sleep quality

Characteristic	Survey participants *N* (%)	Total population *N* (%)	*p*	Migraine *N*, % (95% CI)	Probable migraine *N*, % (95% CI)	Poor sleep quality *N*, % (95% CI) (PSQI > 5)
Sex						
Male	1345 (49.3)	17,584,365 (50.6)	0.854^a^	36, 2.7 (1.8−3.5)	136, 10.1 (8.5−11.8)	334, 24.8 (22.5−27.1)
Female	1350 (50.7)	17,198,350 (49.4)		107, 7.9 (6.5−9.4)	243, 17.9 (15.8−19.9)	381, 28.2 (25.8−30.6
Age, years						
19–29	542 (20.5)	7,717,947 (22.2)	0.917^a^	25, 4.5 (2.7−6.2)	69, 12.6 (9.8−15.4)	153, 28.3 (24.4−32.0)
30–39	604 (21.9)	8,349,487 (24.0)		42, 7.0 (4.9−9.1)	102, 16.8 (13.7−19.8)	136, 22.5 (19.2−25.9)
40–49	611 (23.1)	8,613,110 (24.8)		39, 6.5 (4.5−8.4)	102, 16.8 (13.9−19.8)	167, 27.3 (23.8−30.1)
50–59	529 (18.9)	6,167,505 (17.7)		22, 4.1 (2.4−5.9)	62, 11.6 (8.8−14.4)	160, 30.2 (26.3−34.2)
60–69	409 (15.6)	3,934,666 (11.3)		15, 3.9 (2.0−5.7)	44, 11.2 (8.1−14.2)	99, 24.2 (20.0−28.4)
Size of the residential area						
Large city	1248 (46.3)	16,776,771 (48.2)	0.921^a^	76, 6.1 (4.8−7.5)	180, 14.4 (12.4−16.3)	338, 27.1 (24.6−30.0)
Medium-to-small city	1186 (44.0)	15,164,345 (43.6)		48, 4.0 (2.9−5.2)	174, 14.7 (12.7−16.7)	303, 25.5 (23.1−28.0)
Rural area	261 (9.7)	2,841,599 (8.2)		19, 7.4 (4.2−10.6)	25, 9.7 (6.1−13.3)	74, 28.4 (22.8−33.9)
Education level						
Middle school or less	393 (14.9)	6,608,716 (19.0)	0.752^a^	22, 5.5 (4.2−7.7)	44, 11.5 (8.4−14.7)	110, 28.0 (23.5−32.4)
High school	1208 (44.5)	15,234,829 (43.8)		60, 5.0 (3.8−6.3)	178, 14.7 (12.7−16.7)	317, 26.2 (24.0−28.7)
College or more	1068 (39.6)	12,939,170 (37.2)		60, 5.6 (4.3−7.0)	155, 14.4 (12.3−16.5)	281, 26.3 (23.7−29.0)
Did not respond	26 (9.6)			1, 3.8 (0.0−11.8)	2, 7.7 (0.0−18.7)	7, 26.9 (8.7−45.2)
Total	2695 (100.0)	34,782,715 (100.0)		143, 5.3 (4.5−6.2)	379, 14.1 (12.7−15.4)	715, 26.5 (24.8−28.2)

*N* number, *CI* confidence interval, *PSQI* Pittsburgh Sleep Quality Index

^a^Compared with corresponding value reported in the general population of Korea

### Prevalence of migraine and PM

Of 2695 subjects included in our study, 1273 (47.2%) had at least one headache over the past year, including 143 (5.3%) migraineurs and 379 (14.1%) who had PM. Seven hundred and fifteen (26.5%) participants had poor sleep quality (Table 1). The prevalence of PM was the highest (16.8%) in 30–39 and 40–49 age groups. Of 379 PM participants, 339 (89.5%), 29 (7.7%), and 11 (2.8%) missed criterion B (typical duration of headache), criterion C (typical headache characteristics), and criterion D (typical accompanying symptoms) of code 1.1 migraine diagnostic criteria, respectively.

### Prevalence of poor sleep quality and comparison of PSQI score according to headache diagnosis

The prevalence of poor sleep quality was significantly higher in participants with PM (35.4%) than that in participants with non-headache (21.0%, *p* < 0.001), but lower than that in participants with migraine (47.6%, *p* = 0.011). Regarding PSQI, component scores for latency of sleep (mean ± standard deviation) (1.1 ± 1.0 vs. 0.8 ± 0.9, *p* = 0.001), sleep duration (0.6 ± 0.8 vs. 0.4 ± 0.8, *p* = 0.001), sleep disturbance (0.9 ± 0.6 vs. 0.8 ± 0.5, *p* = 0.001), use of sleeping medication (0.1 ± 0.4 vs. 0.0 ± 0.2, *p* = 0.001), daytime dysfunction (0.8 ± 0.7 vs. 0.5 ± 0.6, *p* = 0.001), and total score (5.2 ± 2.4 vs. 4.2 ± 1.9, *p* = 0.001) were higher in PM participants than those in non-headache participants. However, PSQI component scores for subjective sleep quality (1.7 ± 0.7 vs. 1.7 ± 0.8, *p* = 0.879) and habitual sleep efficacy (0.0 ± 0.3 vs. 0.0 ± 1.5, *p* = 0.119) did not significantly differ between PM and non-headache participants. When comparing PM participants with migraineurs, PSQI component scores for sleep latency and sleep disturbance as well as total PSQI score were higher in participants with migraine than those in participants with PM (Table 2).

**Table 2:** Total and subcomponent PSQI scores among participants with no headache, probable migraine, and migraine

Component	Non-headache *N* = 1422	Probable migraine *N* = 379	Migraine *N* = 143	*p* value
Subjective sleep quality	1.7 ± 0.8	1.7 ± 0.7	1.7 ± 0.7	0.609
Sleep latency	0.8 ± 0.9	1.0 ± 1.0^*^	1.3 ± 1.1^†‡^	< 0.001
Sleep duration	0.4 ± 0.8	0.6 ± 0.8^*^	0.5 ± 0.8^†‡^	0.001
Habitual sleep efficacy	0.0 ± 1.5	0.0 ± 0.3	0.0 ± 0.0	0.116
Sleep disturbance	0.8 ± 0.6	0.9 ± 0.6^*^	1.1 ± 0.6^†‡^	< 0.001
Use of sleeping medication	0.0 ± 0.3	0.1 ± 0.4^*^	0.1 ± 0.5^†^	0.001
Daytime functioning	0.5 ± 0.7	0.8 ± 0.7^*^	0.9 ± 0.9^†‡^	< 0.001
Total	4.2 ± 1.9	5.2 ± 2.4^*^	5.6 ± 2.6^†‡^	< 0.001

*PSQI* Pittsburgh Sleep Quality Index

Data are presented as mean ± standard deviation

Each PSQI component score has a range of 0–3 points. Higher PSQI score indicates more severe disability

^*^p < 0.05 for non-headache vs. probable migraine, by Tukey’s post-hoc analysis

^†^*p* < 0.05 for non-headache vs. migraine, by Tukey’s post-hoc analysis

^‡^*p* < 0.05 for probable migraine vs. migraine, by Tukey’s post-hoc analysis

### Clinical presentations of PM according to presence of poor sleep quality

Participants with PM combined with poor sleep quality had higher proportion of anxiety (*p* < 0.001), depression (*p* < 0.001), and frequency of headache attack (median [interquartile range]: 2.0 [0.3–4.0] vs. 1.0 [0.2–2.0], *p* = 0.001) as well as higher headache intensity (VAS score, 6.0 [4.0–7.0] vs. 5.0 [3.5–6.0], *p* = 0.003) and higher HIT-6 score (50.0 [44.0–58.0] vs. 44.0 [40.0–50.0], *p* = 0.001) compared to participants with PM not combined with poor sleep quality (Table 3). Participants with migraine who combined with poor sleep quality had higher proportion of anxiety, depression, frequency of headache attack and higher HIT-6 score compared to participants with migraine who not combined with poor sleep quality (Table 3).

**Table 3:** Demographics and clinical presentations of participants with migraine and probable migraine stratified according to the presence of poor sleep quality

Characteristic	Migraine with poor sleep quality *N* = 68 (47.5%)	Migraine without poor sleep quality *N* = 75 (52.5%)	*p* value	PM with poor sleep quality *N* = 134 (35.3%)	PM without poor sleep quality *N* = 245 (64.7%)	*p* value
Demographics						
Age, years	41.9 ± 13.0	40.6 ± 11.9	0.546	40.8 ± 13.0	42.5 ± 12.4	0.217
Female	51 (75.0)	56 (74.7)	0.963	86 (64.2)	157 (64.1)	0.985
Headache characteristics						
Bilateral pain	36 (52.9)	45 (60.0)	0.395	73 (54.5)	148 (60.4)	0.263
Non-pulsating quality	46 (67.6)	62 (82.7)	0.037	110 (82.1)	200 (81.6)	0.912
Mild-to-moderate severity	58 (85.3)	57 (76.0)	0.162	12 (9.0)	8 (3.3)	0.018
Not aggravated by movement	48 (70.6)	52 (69.3)	0.870	85 (63.4)	151 (61.6)	0.730
Accompanying symptoms						
Nausea	59 (86.8)	66 (88.0)	0.824	117 (87.3)	20.7 (84.5)	0.456
Vomiting	27 (39.7)	28 (37.3)	0.771	47 (35.1)	68 (27.8)	0.138
Photophobia	41 (60.3)	43 (57.3)	0.719	70 (52.2)	110 (44.9)	0.171
Phonophobia	50 (73.5)	51 (68.0)	0.468	101 (75.4)	167 (68.2)	0.140
Osmophobia	35 (51.5)	33 (44.0)	0.372	71 (53.0)	108 (44.1)	0.097
Accompanying psychiatric problems						
Anxiety (GAS score ≥ 5)	33 (48.5)	10 (13.3)	< 0.001	43 (32.1)	24 (9.8)	< 0.001
Depression (PHQ-9 score ≥ 10)	19 (27.9)	5 (6.7)	0.001	28 (20.9)	5 (2.0)	< 0.001
Headache frequency	2.0 (1.0–7.0)	1.0 (1.0–5.0)	0.009	2.0 (0.3–4.0)	1.00 (0.3–2.0)	0.001
VAS score for headache intensity	7.0 (4.0–10.0)	6.0 (4.0–8.0)	0.247	6.0 (4.0–7.0)	5.00 (3.5–6.0)	0.003
HIT-6 score	57.5 (50.0–65.0)	50.0 (43.0–57.0)	< 0.001	50.0 (44.0–58.0)	44.0 (40.0–50.0)	< 0.001

Data are presented as mean ± standard deviation, number (percent), or median (interquartile range)

*PM* probable migraine, *GAS* Goldberg Anxiety Scale, *PHQ* Patient Health Questionnaire, *VAS* visual analogue scale, *HIT* Headache Impact Test

### Factors associated with PSQI score among PM participants

In multivariable linear regression analysis, anxiety (β = 0.230, *p* < 0.001), depression (β = 0.426, *p* < 0.001), and headache frequency per month (β = 0.090, *p* = 0.031) were significant independent factors associated with total PSQI score in participants with PM (Table 4).

**Table 4:** Analysis^a^ of contributing factors related to the total PSQI score in participants with probable migraine

Factor	Unstandardized coefficients		Standardized coefficients	T	*p* value	Tolerance	VIF
	B	SE	β				
Age	−0.086	0.085	−0.046	−1.019	0.309	0.810	1.235
Sex	−0.177	0.202	−0.036	−0.877	0.381	0.969	1.032
Size of residential area	−0.086	0.157	−0.022	− 0.548	0.584	0.988	1.012
Educational level	−0.082	0.131	−0.028	−0.629	0.529	0.792	1.262
Anxiety (GAS score ≥ 5)	0.231	0.052	0.230	4.438	< 0.001	0.602	1.660
Depression (PHQ-9 score ≥ 10)	0.230	0.027	0.426	8.471	< 0.001	0.640	1.563
Headache frequency per month	0.039	0.018	0.090	2.166	0.031	0.928	1.077
VAS score for headache intensity	0.088	0.056	0.065	1.556	0.121	0.923	1.083

*PSQI* Pittsburgh Sleep Quality Index, *SE* standard error, *VIF* variation inflation factor, *GAS* Goldberg Anxiety Scale, *PHQ* Patient Health Questionnaire, *VAS* visual analogue scale

Independent variables included sociodemographic variables (age, sex, size of residential areas, and education level), anxiety (GAS score ≥ 5), depression (PHQ-9 score ≥ 10), headache frequency per month, and VAS score for headache intensity, whereas total PSQI score was included as the dependent variable

^a^multivariate linear regression: R^2^ = 0.194, adjusted R^2^ = 0.139

## Discussion

The main findings of our research were as follows. First, prevalence of migraine, PM, and poor sleep quality in Korean general population were 5.3%, 14.1%, and 26.5%, respectively. Second, 35.3% of participants with PM had poor sleep quality, which was lower than the prevalence noted in participants with migraine, but higher than that in participants with non-headache. Third, among PM participants, those with poor sleep quality showed increased headache frequency, intensity, and impact.

Among migraineurs, poor sleep quality is not an uncommon problem. A clinic-based study in India has reported that 66.7% of migraineurs without aura show poor sleep quality. This proportion is higher than that in non-migraine participants [14]. A Chinese study investigating 1023 nurses has demonstrated that, compared to tension-type headache or non-headache participants, migraineurs have reported significantly higher frequency of poor sleep quality [27]. In our study, the first to assess the prevalence of poor sleep quality among individuals with PM in a population-based setting, approximately half of migraineurs and one-third of individuals with PM experienced poor sleep quality. This confirms that poor sleep quality is a common comorbidity not only in migraineurs, but also in individuals with PM.

Among PSQI components, latency of sleep, duration of sleep, sleep disturbance, hypnotics use, and daytime dysfunction scores were significantly higher in participants with PM than those in non-headache participants. This suggests that various aspects of sleep quality are impaired in individuals with PM. One interesting finding is the impairment of daytime functioning among participants with PM. Indeed, individuals with migraine or PM often report disability even during interictal period [28]. Migraineurs are less physically active with reduced vigour. They show higher levels of sleepiness and more anxiety and avoidance [28]. Our findings are in line with results of previous observation [28], additionally indicating that daytime dysfunction in PM patients might be associated with poor sleep quality.

Our study demonstrated that anxiety, depression, and headache frequency were associated with poor sleep quality. This is in agreement with previous observations showing that anxiety and depression are independently related factors for PSQI score [29, 30] and that headache frequency is positively associated with PSQI score among migraineurs [31]. Our results confirmed that anxiety, depression, and headache frequency were significant factors for PSQI score among individuals with PM.

Insomnia is a prevalent sleep disorder. Individuals with insomnia may have difficulty falling asleep, staying asleep or early awakening even if enough time is given [32]. Poor sleep quality can occur as a result of insomnia [33]. Nevertheless, it can be caused by other conditions such as sleep apnea, shift working, use of medications, environmental factors et al. [34–37]. Therefore, the poor sleep quality is related to insomnia but measures border sleep difficulties. We already investigated the association of poor sleep quality and migraine in a population-based sample [38]. Here, we firstly report the relationship of poor sleep quality and PM, another common and disabling headache disorder.

Based on findings of the present study, we propose the following strategies to improve sleep quality in participants with PM. Since anxiety, depression, and headache frequency are independent factors associated with poor sleep quality in individuals with PM, they could be successfully managed by pharmacological and non-pharmacological treatments. Anxiolytic drugs and antidepressants are effective in reducing anxiety and depression symptoms [39]. Non-pharmacological cognitive behaviour therapy (CBT) can be used to treat anxiety and depression [40]. Headache frequency can be reduced by preventive pharmacological treatment and non-pharmacological treatments such as relaxation techniques, CBT, education, and mindfulness [41]. Therefore, pharmacological and non-pharmacological treatments of anxiety, depression, and headache frequency might be able to improve sleep quality of individuals with PM. Such strategies may improve symptoms of PM by improving sleep quality.

In the present study, PM participants with poor sleep quality had more headache frequency and more severe headaches than PM participants without poor sleep quality. Considering that headache frequency and intensity are closely associated with headache-related disability and health-related quality of life, poor sleep quality may be an important factor for such aspects in individuals with PM [42]. Sleep quality may be improved using pharmacological and non-pharmacological treatments [43]. Our present findings suggest that, among individuals with PM, proper assessment and treatment of poor sleep quality may be needed to reduce headache-related disability and improve quality of life besides improving headache symptoms.

Our research has some limitations. First, although questionnaire was utilized to the diagnosis of PM and migraine, this questionnaire was only validated in migraine, but not validated in PM. According to ICHD-2, the diagnosis of PM is diagnosed only when one of the criteria of migraine is not satisfied. Thus, validation for PM itself is not necessarily required. Second, we evaluated sleep quality using a questionnaire without performing actigraphy or polysomnography to confirm the sleep quality objectively. Nevertheless, we found it not only practical, but also appropriate to use PSQI because this tool was validated for assessing sleep quality, showing good agreement with polysomnography and actigraphy measurements [44]. Therefore, it has been widely adopted in clinical and epidemiological studies. Third, although our study was population-based with low sampling error, its statistical power was limited because our study could not preform subgroup analysis due to limited sample size. Lastly, we did not included medications for migraine and PM treatment in analyses. Although a significant proportion of migraineurs did not receive medical treatment, some medications for acute and preventive treatments may influence on sleep quality. Caffeine, a common ingredient of acute migraine treatment, may impair sleep quality [45]. Topiramate is a widely used medication for preventive migraine treatment and may induce dysphoria or unmask latent mood disorder [46]. Further studies are needed the effect of migraine medications on sleep quality among individuals of migraine and PM.

On the other hand, the present study has several strengths. First, our study used a questionnaire whose Korean version was specifically validated for assessing migraine, anxiety, depression, and sleep quality. Second, we applied clustered random sampling proportional to the distribution in the Korean general population with low sampling error which allowed us to accurately assess the prevalence of migraine, PM, and poor sleep quality. Third, we investigated PSQI component scores in addition to total scores. PSQI comprises seven components including subjective sleep quality, latency of sleep, duration of sleep, habitual sleep efficacy, sleep disturbance, use of hypnotics, and dysfunction at daytime. By investigating these various aspects of sleep quality separately, we were able to determine that some components were impaired in participants with PM.

## Conclusion

Although the prevalence of poor sleep quality was lower in participants with PM than that in migraineurs, prevalence remained high in those with PM. It was associated with worse symptoms of PM. Our results suggest that appropriate diagnostic approach and management of poor sleep quality are necessary to manage PM appropriately.

## Additional file

Additional file 1: Raw data of the present study. (XLS 1035 kb)

## Abbreviations

HIT-6: Headache impact test-6
ICHD: International classification of headache disorders
KHSS: Korean headache-sleep study
PHQ-9: Patient health questionnaire-9
PM: Probable migraine
PSQI: Pittsburgh sleep quality index
VAS: Visual analogue scale
